# Supplementary material for: Identification of photoperiod-induced specific miRNAs in the adrenal glands of Sunite sheep (Ovis aries)
Source: Front Vet Sci. 2022 Jul 22;9:888207. doi: 10.3389/fvets.2022.888207 (PMC9354845; doi:10.3389/fvets.2022.888207)
Supplement: Supplementary file 1 [file Data_Sheet_1.ZIP › Supplementary file/miRDeep2 file.zip!/miRDeep2 file/Know_Structure/SPLP42A3/oar-mir-3955.pdf]

miRBase precursor : oar-mir-3955
Total read count : 431
oar-miR-3955-5p read count : 379
oar-miR-3955-3p read count : 35
remaining reads : 17

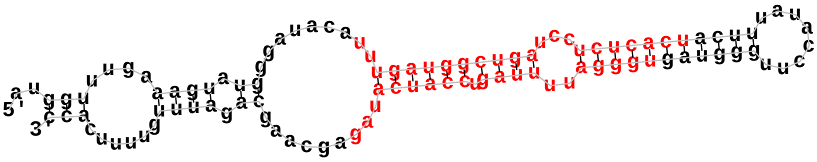

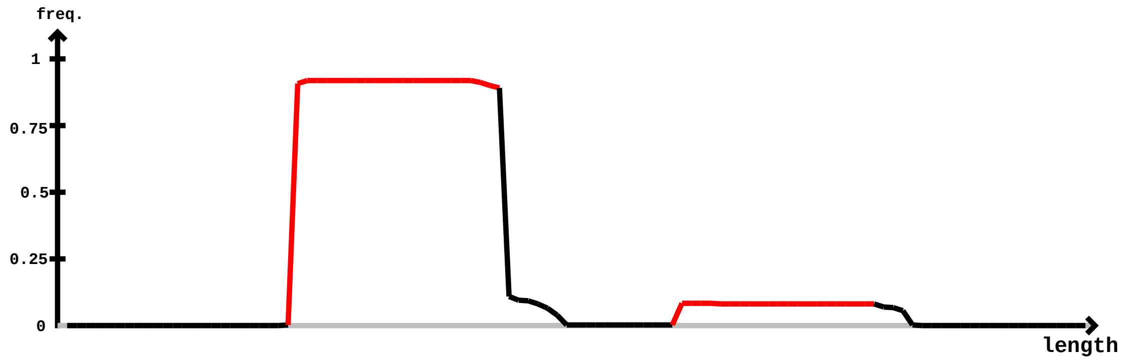


```
                                               oar-miR-3955-5p
                                                                                            oar-miR-3955-3p
5'- augguuugaaaguaugggauacauuugauggcugauccucucacuacuuuauaccuuggguagugggauuuuaguccaucauagagcaagcagauuuguuuucacc   -3'    exp
    ...........((((...))))...((((((((((...(((((((((((........)))))))))))..)))).)))))).(((((((......)))))))....    reads      mm         sample
    ......................auuugauggcugauccucuc................................................................    1          0          seq
    .......................uuugauggcugauccucuc................................................................    2          0          seq
    .......................uuugauggcugauccucuca...............................................................    5          0          seq
    .......................uuugauggcugauccucucac..............................................................    4          0          seq
    .......................uuugauggcugauccucucacu.............................................................    332        0          seq
    .......................uuugauggcugauccucucacua............................................................    6          0          seq
    .......................uuugauggcugauccucucacuac...........................................................    1          0          seq
    .......................uuugauggcugauccucucacuacu..........................................................    5          0          seq
    .......................uuugauggcugauccucucacuacuu.........................................................    7          0          seq
    .......................uuugauggcugauccucucacuacuuu........................................................    11         0          seq
    .......................uuugauggcugauccucucacuacuuua.......................................................    16         0          seq
    .......................uuugauggcugauccucucacuacuuuauaccuuggguagugggg........................................    1          0          seq
    ........................uugauggcugauccucucacu.............................................................    5          0          seq
    ...............................................................ugggauuuuaguccaucauag......................    5          0          seq
    ...............................................................ugggauuuuaguccaucauaga.....................    1          0          seq
    ...............................................................ugggauuuuaguccaucauagag....................    5          0          seq
    ...............................................................ugggauuuuaguccaucauagagc...................    23         0          seq
    ...............................................................ugggauuuuaguccaucauagagca..................    1          0          seq
```
